# Ionic Liquid-Promoted Three-Component Domino Reaction of Propargyl Alcohols, Carbon Dioxide and 2-Aminoethanols: A Thermodynamically Favorable Synthesis of 2-Oxazolidinones

**DOI:** 10.3390/molecules23113033

**Published:** 2018-11-20

**Authors:** Shumei Xia, Yu Song, Xuedong Li, Hongru Li, Liang-Nian He

**Affiliations:** 1 State Key Laboratory and Institute of Elemento-Organic Chemistry, College of Chemistry, Nankai University, Tianjin 300071, China; 2120160737@mail.nankai.edu.cn (S.M.); songyu@mail.nankai.edu.cn (Y.S.); xuedong-li@163.com (X.L.); 2 College of Pharmacy, Nankai University, Tianjin 300353, China

**Keywords:** ionic liquid, synergistic activation, aminoethanol, 2-oxazolidinone, atmospheric CO_2_, sustainable catalysis

## Abstract

To circumvent the thermodynamic limitation of the synthesis of oxazolidinones starting from 2-aminoethanols and CO_2_ and realize incorporation CO_2_ under atmospheric pressure, a protic ionic liquid-facilitated three-component reaction of propargyl alcohols, CO_2_ and 2-aminoethanols was developed to produce 2-oxazolidinones along with equal amount of α-hydroxyl ketones. The ionic liquid structure, reaction temperature and reaction time were in detail investigated. And 15 mol% 1,5,7-triazabicylo[4.4.0]dec-5-ene ([TBDH][TFE]) trifluoroethanol was found to be able to synergistically activate the substrate and CO_2_, thus catalyzing this cascade reaction under atmospheric CO_2_ pressure. By employing this task-specific ionic liquid as sustainable catalyst, 2-aminoethanols with different substituents were successfully transformed to 2-oxazolidinones with moderate to excellent yield after 12 h at 80 °C.

## 1. Introduction

The accumulation of CO_2_ in atmosphere has aroused considerable concern due to its link to the climate change; hence effective strategies are urgently needed to mitigate the increasing CO_2_ buildup [1,2,3]. Among the various strategies, transforming CO_2_ to valuable chemicals has received much attention by utilizing CO_2_ as economic and nontoxic C_1_ source [4,5]. Nowadays, CO_2_ has been employed in the synthesis of carboxylic acids [6,7,8], carbonates [9,10,11,12], carbamates [13,14,15,16,17,18], formate [19,20,21], methanol [21,22,23,24], ureas [25,26,27] and polymers [28,29,30] as well.

**Scheme 1:**
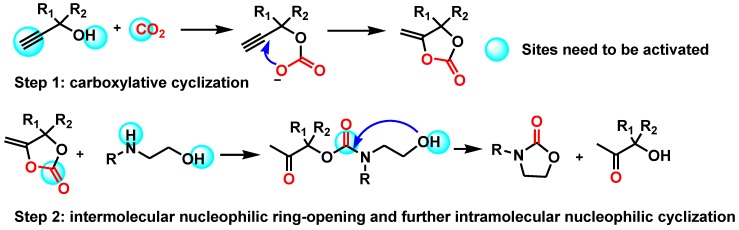
Stepwise reaction in the three-component domino reaction and sites need to be activated.

Oxazolidinones, which are in the family of carbamates, are important chiral auxiliaries in organic synthesis [31,32] and are also common structural units in drugs [33,34] and agrochemicals [35]. However, the conventional synthetic methods of 2-oxazolidinones rely on hazardous or expensive reagents such as isocyanides [36] and phosgene [37]. Recently, B. Gabriele et al. reported an alternative unconventional synthetic method to produce oxazolidinones starting from 2-amino alcohols with CO in ionic liquids [38]. As the surrogate of CO, CO_2_ is a more desirable material. Therefore, synthesis of 2-oxazolidinones using CO_2_ and appropriate substrates has attracted much attention [14,39,40], among which the direct synthesis of oxazolidinones starting from 2-aminoethanols and CO_2_ is considered as an ideal route because the aqueous amino alcohols have been widely utilized to capture CO_2_ in industry [41,42]. Unfortunately, the direct reaction between amino alcohols and CO_2_ occurs difficultly due to the thermodynamic limitation [43]. To shift the equilibrium to 2-oxazolidinones, harsh reaction conditions [44,45,46,47,48], dehydrating agents or auxiliaries are often needed [13,49,50,51,52]; thus, the generation of wasteful by-products is unavoidable. As an alternative strategy, our group has introduced propargyl alcohols into the reaction system of amino alcohols and CO_2_ to circumvent this thermodynamic limitation [53,54,55]. As descripted in Scheme 1, this three-component cascade reaction is composed of carboxylative cyclization and the following intermolecular nucleophilic ring-opening and further intramolecular nucleophilic cyclization. Although Ag- and Cu-based catalysts have been developed for this three-component reaction to coproduce 2-oxazolidinones and α-hydroxyl ketones, high CO_2_ pressure is still needed. In this context, efficient catalyst is still highly desirable to run this reaction at atmospheric pressure.

It is speculated the synergistic activation of the substrate and CO_2_ may facilitate this tandem reaction running at atmospheric pressure. Promisingly, the ionic liquids (ILs) show the potential of multi-site activation in many organic syntheses by choice or functionalization of cations or anions [56,57,58,59]. Especially, the ILs-promoted carboxylative cyclization of propargyl alcohols and CO_2_ has been reported [60]. Considering this carboxylative cyclization reaction is the crucial step in this three-component cascade reaction of propargyl alcohols, amino alcohols and CO_2_, we envisioned that the ILs-catalyzed three-component reaction is feasible.

Herein, a task-specific ILs-catalyzed three-component reaction of propargyl alcohols, CO_2_ and 2-aminoethanols was reported. By subtly selecting the cation and anion of the ionic liquid, 1,5,7-triazabicylo[4.4.0]dec-5-ene trifluoroethanol ([TBDH][TFE]) was found to be the most suitable catalyst and excellent yield of 2-oxazolidinones and α-hydroxyl ketones could be obtained at atmospheric CO_2_ pressure.

## 2. Results and Discussion

### 2.1. Optimization of the Reaction Conditions

The initial attempt to perform the three-component domino reaction of 2-(benzylamino)ethanol (**1a**), 2-methylbut-3-yn-2-ol (**2a**) and CO_2_ using ILs as catalyst was carried out under 1 atm CO_2_ at 80 °C for 12 h, as summarized in Table 1. Considering the anion may play an important role in activation of the substrate **2a**, **1a** and CO_2_, the anions of ILs were firstly screened. Thus, five 1,8-diazabicyclo[5.4.0]undec-7-ene (DBU) (1,5,7-triazabicylo[4.4.0]dec-5-ene)-based ILs with different anions were investigated for this purpose (entries 1–5). As a result, the IL with anion [TFE]^−^ presented the highest activity with 23% yield of the target product **3a** (3-benzyl oxazoline-2-ketone) and 27% yield of **4a** (3-hydroxyl-3-methyl butanone) (entry 3,), which is attributed to the activation capacity of [TFE]^−^ to CO_2_ [61] and to **1a** as well, being consistent with the reaction mechanism as depicted in Scheme 1. On the other hand, the cation of IL is supposed to activate the triple bond of **2a** and also carbonyl group of the intermediate i.e., *α*-alkylidene cyclic carbonate, thus facilitates the carboxylative cyclization of **2a** and the following intermolecular nucleophilic ring-opening and further intramolecular nucleophilic cyclization. Therefore, a variety of cations, including [DBUH]^+^, [TBDH]^+^, [TMGH]^+^ and [P_4444_]^+^, were further evaluated with [Im]^−^ as the anion (entries 1,6–8), and [TBDH]^+^ was found to be the most effective cation, which may be related with its stronger interaction with the substrate **2a** and the intermediate. The above findings showed that both the anions and cations can affect the the catalytic ability of the ILs in this reaction. We speculated that the IL with the combination of [TBDH]^+^ and [TFE]^−^ would be an efficient catalyst for this domino reaction. As expected, the yield of the products was improved, and 33% yield of **3a** and 39% of **4a** were obtained when [TBDH][TFE] was used as the catalyst (entry 9).

Considering the IL amount may also affect the catalytic capability, the amount of the IL was increased to further improve the reaction (Table 2). Surprisingly, the reduction of yields was observed for most of the ionic liquids when the amount of the tested IL was increased from 5 mol% to 10 mol% (entries 1–6). It is inferred that the interaction between the cations of ILs and the nucleophilic sites of the substrate became dominant with the increase of the amount of the IL, which can deactivate these nucleophilic sites and thus impedes the domino reaction. Conversely, increasing the amount of [TMGH][Im], [P_4444_][Im] and [TBDH][TFE] would promote the reaction (entries 7–9), wherein [TBDH][TFE] showed higher activity than other ionic liquids used in this study. With [TBDH][TFE] as catalyst, the reaction time and reaction temperature were further studied. The results showed that extending the reaction time (entry 10) or raising the reaction temperature (entries 11,12) had no obvious impact on the reaction results. But if the loading of [TBDH][TFE] was increased to 15 mol%, the **3a** yield of up to 94% was obtained (entry 13). Further improving the loading of [TBDH][TFE] to 20 mol% reduced the yield greatly (entry 14), which may be attributed to the solvent effect of the IL and also the deactivation of the nucleophilic sites by [TBDH]^+^. No target product was obtained without any catalyst or with only TBD or TFE as catalyst (entries 15–17), which indicates the importance of free anion and cation in activating substrate and CO_2_. Therefore, 15 mol% [TBDH][TFE] was used as catalyst and the reactions were performed at 80 °C for 12 h in subsequent studies.

### 2.2. Investigation of Substrate Applicability

Having selected suitable IL as catalyst, we proceeded to investigate the substrate applicability as listed in Table 3. The propargyl alcohols, 2-aminoalcohols and even glycols with different substituents were employed in this cascade reaction. A wide range of propargyl alcohols with methyl, ethyl, cyclohexyl, aryl and even other complicated substitute at the propargylic position (**2a**–**e**), could be transformed effectively to afford the corresponding 2-oxazolidinones and *α*-hydroxyl ketones in moderate to high yields (entries 1–5). Notably, this protocol showed its potential application in pharmaceutical industry by realizing the transformation of ethisterone (**2e**) to *α*-hydroxyprogesterone (**4e**) with 63% of yield and the chiral structure retained (entry 5). Subsequently, the suitability of 2-aminoalcohols with different substitutes was further examined (entries 6–11). Both aryl-substituted and alkyl-substituted 2-aminoalcohols proceeded smoothly and generated the corresponding products in excellent yields with the exception of aromatic 2-aminoalcohols bearing nitro group at *para*-position (entry 10). The results may be interpreted by the weakened nucleophilicity of amino caused by the strong electron-withdrawing nitro group at benzene ring [62,63]. Besides 2-aminoalcohols, glycols (**1h**–**j**) were also converted to the corresponding cyclic carbonates in moderate to good yields (entries 12–14).

### 2.3. Recycle Tests

**Figure 1:**
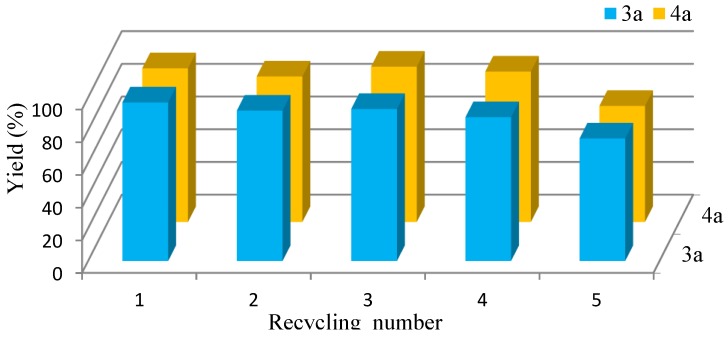
Reusability of the [TBDH][TFE] system. Reaction conditions: **1a** (302.4 mg, 2.0 mmol), **2a** (168.2 mg, 2.0 mmol), [TBDH][TFE] (71.8 mg, 15 mol%), CO_2_ (1 atm), 80 °C, 12 h.

As ILs are non-volatile and thermal stable, they can be easily recovered by evaporating the low boiling point components. So, we subsequently explored the recycle performance of [TBDH][TFE] for this tandem reaction using **1a** and **2a** as model substrates under the optimum conditions. After reaction, the gas was released and the reaction system was subjected to vacuum distillation for at least 1 h at 180 °C. After removing the reactant and product, the catalyst was directly used in the next round of the reaction. As depicted in Figure 1, the IL can be reused without considerable activity loss for at least four times. The target product could still be obtained at a 75% yield after the catalyst was reused for 5 times, suggesting that the system has a good recyclability (as shown in Figure 1).

### 2.4. Mechanism Study

According to our early reports [54,55], this cascade reaction is composed of carboxylative cyclization and the following intermolecular nucleophilic ring-opening and further intramolecular nucleophilic cyclization. To gain deeper insight on the role of [TBDH][TFE] in the catalysis, the stepwise reactions were conducted (Table 4). To begin with, we performed the carboxylative cyclization of 2-methylbut-3-yn-2-ol (**2a**) with CO_2_ using 10 mol% of IL as catalyst. A variety of DBU-based ILs such as [DBUH][Im]), [DBUH][OAc], [DBUH][Cl], and Im-based ILs including [TBDH][Im], [TMGH][Im] showed high efficiency to produce 4,4-dimethyl-5-methylene-1,3-dioxolan-2-one (**m**) with excellent yield under mild conditions (entries 1–2,5–7). In contrast, [TBDH][TFE] and [DBUH][TFE] showed lower activity in the carboxylative cyclization reaction, probably due to the weaker hydrogen bond receptors of anions (entries 3,4,9, *vs.* 1–2,5–7) [64,65,66]. The same reason may be used to explain the low activity of [DBUH][TFA]. For [P_4444_][Im] (tetra butylphosphonium imidazolide), the large steric hindrance may impede the interaction of cation and carbon-carbon triple bond and thus the substrate can’t be activated efficiently (entry 8).

The activity of different ILs on the subsequent reaction of vinyl cyclic carbonate (**m**) and aminoethanol (**1a**) was further examined under the otherwise identical conditions (Table 3). Noticeably, [DBUH][Im], [DBUH][OAc] and [TBDH][TFE] exhibited similar catalytic behavior to afford quantitative yield of **3a** and **4a** (entries 1,2,9, Table 3), while other ILs showed moderate activities (entries 3–8, Table 3).

The interesting results can be obtained by comparing the results of stepwise reaction with three-component cascade reaction. Most ionic liquids, which can promote the stepwise reaction, display reduced performance in the three-component reaction (Table 2 and Table 4). Depressingly, the reason for the decreased catalytic activity of ILs in the three-component reaction is not clear at this stage. Fortunately, the catalytic activity of [TBDH][TFE] is retained in the three-component reaction and its catalytic performance can be improved by increasing the loading amount from 10 mol% to 15 mol% (Table 2). It is inferred that *α*-alkylidene cyclic carbonate is the key intermediate in this multistep cascade reaction [53,54,55]. Although [TBDH][TFE] was not so efficient in the carboxylative cyclization, we speculated that the added ethanolamine can act as a base to facilitate the activation of hydroxyl in propargyl alcohol, thus efficiently promoting carboxylative cyclization of 2-methylbut-3-yn-2-ol (**2a**) with CO_2_. To verify our conjecture, ^1^H NMR (400 MHz, DMSO-d6) measurement was performed to characterize the interaction between propargyl alcohol and 2-aminoalcohol. The results showed that the peak shape for hydroxylic hydrogen in 2-methylbut-3-yn-2-ol (**2a**) altered after addition of 2-(benzylamino)ethanol (**1a**), proving the formation of hydrogen bond between propargyl alcohol and 2-aminoalcohol (see Figure S1 in Supplementary Materials).

**Scheme 2:**
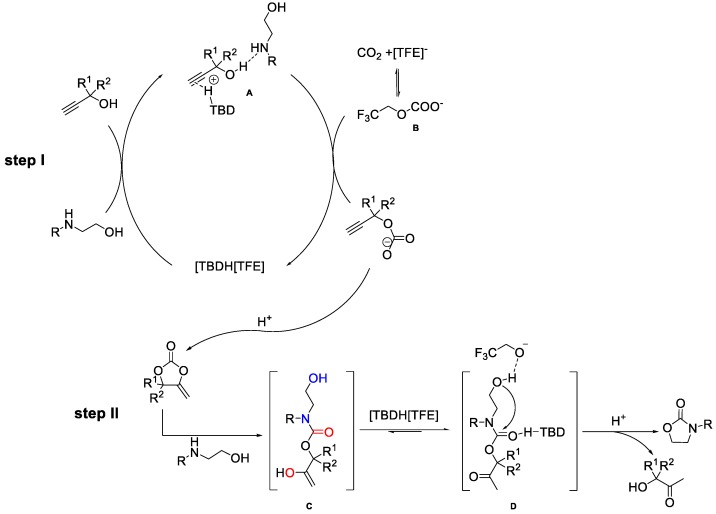
Plausible mechanism.

On the basis of the experimental studies and previous reports [54], the possible mechanism for the [TBDH][TFE]-catalyzed three-component reaction was proposed as depicted in Scheme 2. This cascade reaction includes the initial carboxylative cyclization of 2-methylbut-3-yn-2-ol (**2a**) with CO_2_ and the subsequent nucleophilic addition of *α*-alkylidene cyclic carbonate with 2-aminoalcohol. In step **I**, propargyl alcohol is simultaneously activated by the coordination of the C≡C bond with cation [TBDH]^+^ and hydrogen bonding with 2-aminoethanol to form the intermediate **A**, while CO_2_ is activated through the formation of the anion [TFECOO]^−^. Then the α-alkylidene cyclic carbonate intermediate is generated by the nucleophilic attack of O atom in the intermediate **A** to CO_2_ and subsequent intramolecular cyclization. In step **II**, the nucleophilic N atom of 2-aminoethanol attacks α-alkylidene cyclic carbonate to form the corresponding *β*-oxopropylcarbamate species C [54]. Finally, 2-oxazolidinones and equal amount of *α*-hydroxyl ketones are formed through the intramolecular cyclization of intermediate C facilitated by [TBDH]^+^.

## 3. Materials and Methods

### 3.1. Materials and General Analytic Methods

Unless otherwise noted, carbon dioxide (99.999%), commercially available DBU (99%), TBD (98%), TMG (99%), Imidazole (99.5%), TFE (99.5%) and TFA (99.5%) were obtained from Shanghai Aladdin Bio-Chem Technology Co., LTD. (Shanghai, China). All starting materials including propargyl alcohols, monoethanolamine and aromatic aldehydes, were obtained from Aladdin, Alfa Aesar (Haverhill, MA, USA), and Heowns (Tianjin, China) and used as received. All operations were carried out by using standard high vacuum and Schlenk technique unless otherwise noted. ^1^H NMR spectra were recorded on Bruker AVANCE III HD 400 spectrometer (Bruker BioSpin, Rheinstetten, Germany) using CDCl_3_ (99.8%, Wilmad, Beijing, China) or DMSO-d*6* (99.9%, Wilmad, Beijing, China) as solvent referenced to CDCl_3_ (7.26 ppm) or DMSO-d6 (2.50 ppm). ^13^C NMR was recorded at 100.6 MHz in CDCl_3_ (77.00 ppm). Multiples were assigned as singlet, doublet, triplet, doublet of doublet, multiplet and broad singlet. Mass spectra were recorded on a Shimadzu GCMS-QP2010 (Restek, Shimadzu, Japan) equipped with a RTX-5MS capillary column (Restek, Shimadzu, Japan) at an ionization voltage of 70 eV. The data are given as mass units per charge (m/z).

### 3.2. General Procedure for the Synthesis of 2-Aminoethanol Derivatives [33]

A mixture of an aromatic aldehyde (10 mmol), 2-aminoethanol (0.61 g, 10 mmol) and Na_2_SO_4_ (1.42 g, 10 mmol) in CH_3_OH (20.0 mL) was added into a 100 mL round bottom flask and stirred for 2 h at room temperature. Then, the solution was obtained after removing the drying agent. The solution was treated with the addition of NaBH_4_ (0.19 g, 5 mmol) in ice water and stirred for 1 h. The mixture was concentrated under vacuum, and the residue was extracted with CH_2_Cl_2_ (3 × 20 mL), washed with water (20.0 mL), and dried with Na_2_SO_4_. The CH_2_Cl_2_ solution was concentrated under vacuum and the residue was purified by column chromatography on silica gel using petroleum ether/ethyl acetate (*v/v*, 10:1–1:2) as eluent to give the desired products.

### 3.3. General Procedure for the Synthesis of ILs [67]


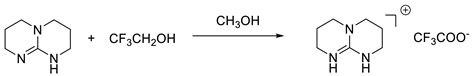


All ionic liquids (ILs) were synthesized based on the procedure’s reported [59]. Typically, for the synthesis of [TBDH][TFE], TBD was added to the methanol solution of CF_3_CH_2_OH by using the anion-exchange resin method. Then the mixture was stirred at room temperature for 24 h. Subsequently, methanol was distilled off at 70 °C under vacuum for 24 h. The as-synthesized ILs were characterized by NMR spectroscopy. The NMR data are listed in Supplementary Material.

### 3.4. General Procedure for the Synthesis of 2-Oxazolidinones and α-Hydroxyl Ketones

[TBDH][TFE] (69.9 mg, 15 mol%), propargylic alcohol **1a** (2.0 mmol) and 2-aminoethanol (2.0 mmol) were added successively to a 10 mL Schlenk tube equipped with a magnetic stir bar. Then the flask was capped and attached to a CO_2_ balloon with purity of 99.95%. Subsequently, the reaction mixture was stirred at 80 °C for 12 h. When the reaction completed, the vessel was cooled with an ice-bath, and remove the balloon. The residue was flushed with 2 × 3 mL CH_2_Cl_2_ and purified by column chromatography on silica gel (*n*-butylamine saturation) using petroleum ether/ethyl acetate (*v/v*, 50:1–1:1) as eluent to give the desired products.

## 4. Conclusions

In summary, we have developed a new strategy to promote the three-component cascade reaction of propargyl alcohols, atmospheric CO_2_ and 2-aminoethanols efficiently under mild conditions by utilizing ILs as both catalyst and solvent, which successfully addresses the thermodynamic issue in the synthesis of 2-oxazolidinones starting from 2-aminoethanols with CO_2_ and avoids the generation of wasteful by-products. The protic IL [TBDH][TFE] showed the best performance amongst the tested ILs due to its ability to synergistically activate the substrate by its cation and anion. Besides, it can be easily recovered and reused at least for 5 times without obvious loss of its activity. A wide range of propargyl alcohols and 2-aminoethanols, were successfully transformed to 2-oxazolidinones and α-hydroxyl ketones with moderate to excellent yield after 12 h at 80 °C. We believe that employing easily recyclable ILs as catalyst and CO_2_ as starting material renders the synthesis of 2-oxazolidinones in sustainable mode. 

## Figures and Tables

**Table 1:**
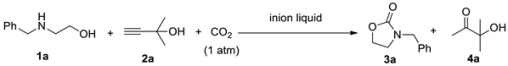
Screening ILs for the three-component domino reaction ^[a]^.

Entry	IL (mol%)	Yield/% ^[b]^	
		3a	4a
1	[DBUH][Im]	14	13
2	[DBUH][OAc]	19	21
3	[DBUH][TFE]	23	27
4	[DBUH][TFA]	21	21
5	[DBUH][Cl]	13	9
6	[TBDH][Im]	27	30
7	[TMGH][Im]	22	21
8	[P_4444_][Im]	trace	trace
9	[TBDH][TFE]	33	39

^[a]^ Unless otherwise specified, the reaction conditions were: **1a** (302.4 mg, 2.0 mmol), **2a** (168.2 mg, 2.0 mmol), IL (5 mol%), CO_2_ (1 atm), 80 °C, 12 h. ^[b]^ Determined by ^1^H-NMR with 1,1,2,2-tetrachloroethane as the internal standard. DBU = 1,8-diazabicyclo[5.4.0]undec-7-ene, [DBUH][Im] = DBU imidazolide, [DBUH][OAc] = DBU acetate, [DBUH][TFE] = DBU trifluoroethanol, [DBUH][TFA] = DBU trifluoracetic acid, [DBUH]Cl = DBU chloride, [TBDH][Im] = 1,5,7-Triazabicylo[4.4.0]dec-5-ene imidazolide, [TMGH][Im] = 1,1,3,3-tetramethylguanidinium imidazolide, [P_4444_][Im] = hexyltributylphosphonium imidazolide, [TBDH][TFE] = 1,5,7-Triazabicylo[4.4.0]dec-5-ene trifluoroethanol.

**Table 2:** Optimization of the reaction conditions ^[a]^.

Entry	IL (mol%)	Yield/% ^[b]^	
		3a	4a
1	[DBUH][Im] (10)	13	11
2	[DBUH][OAc] (10)	7	6
3	[DBUH][TFE] (10)	3	5
4	[DBUH][TFA] (10)	9	9
5	[DBUH]Cl (10)	13	16
6	[TBDH][Im] (10)	5	9
7	[TMGH][Im] (10)	33	37
8	[P_4444_][Im] (10)	25	26
9	[TBDH][TFE] (10)	59	55
10 ^[c]^	[TBDH][TFE] (10)	57	58
11 ^[d]^	[TBDH][TFE] (10)	47	47
12 ^[e]^	[TBDH][TFE] (10)	56	53
13	[TBDH][TFE] (15)	94	97
14	[TBDH][TFE] (20)	44	46
15	TBD (15)	trace	trace
16	TFE (15)	trace	trace
17	—	trace	trace

^[a]^ Reactions were carried out at 80 °C for 12 h with **1a** (302.4 mg, 2.0 mmol), **2a** (168.2 mg, 2.0 mmol) and CO_2_ (1 atm). ^[b]^ Determined by ^1^H-NMR with 1,1,2,2-tetrachloroethane as the internal standard. ^[c]^ 18 h. ^[d]^ 60 °C. ^[e]^ 100 °C.

**Table 3:**
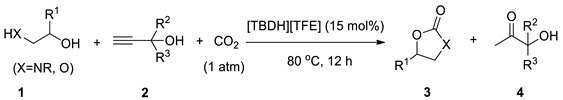
Scope of the substrates for the reaction under optimized conditions ^[a]^.

Entry	Substrate		Yield/% ^[b]^	
	1	2	3	4
1	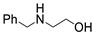 **1a**	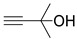 **2a**	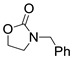 **3a** (94, 90^c^)	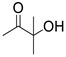 **4a** (97, 93^c^)
2	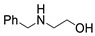 **1a**	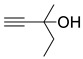 **2b**	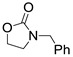 **3a** (80)	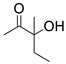 **4b** (79)
3	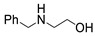 **1a**	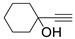 **2c**	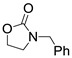 **3a** (73)	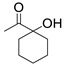 **4c** (76)
4	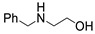 **1a**	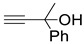 **2d**	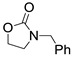 **3a** (99)	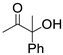 **4d** (99)
5	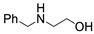 **1a**	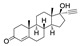 **2e**	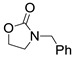 **3a** (61)	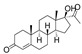 **4e** (63)
6	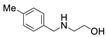 **1b**	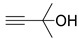 **2a**	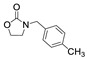 **3b** (89)	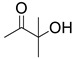 **4a** (89)
7	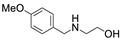 **1c**	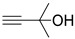 **2a**	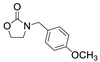 **3c** (85)	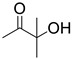 **4a** (82)
8	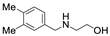 **1d**	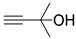 **2a**	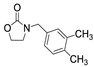 **3d** (99)	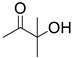 **4a** (99)
9	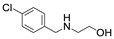 **1e**	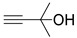 **2a**	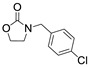 **3e** (82)	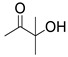 **4a** (80)
10	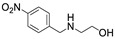 **1f**	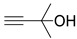 **2a**	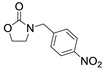 **3f** (24)	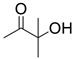 **4a** (23)
11	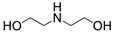 **1g**	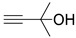 **2a**	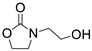 **3g** (93)	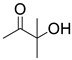 **4a** (92)
12	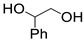 **1h**	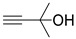 **2a**	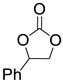 **3h** (95)	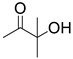 **4a** (97)
13	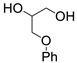 **1i**	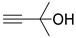 **2a**	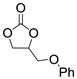 **3i** (58)	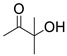 **4a** (57)
14	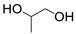 **1j**	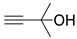 **2a**	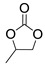 **3j** (94)	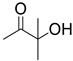 **4a** (98)

^[a]^ Reactions were performed using **1** (2.0 mmol), **2** (, 2.0 mmol), [TBDH][TFE] (71.8 mg, 15 mol%) with CO_2_ pressure of 1 atm at 80 °C for 12 h. ^[b]^ Determined by ^1^H-NMR with 1,1,2,2-tetrachloroethane as the internal standard. ^[c]^ Isolated yield.

**Table 4:**
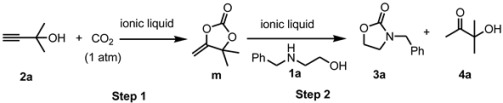
Catalytic effect of ionic liquids to stepwise reactions in the domino reaction ^[a]^.

Entry	IL	Yield/% ^[b]^		
		m	3a	4a
1	[DBUH][Im]	87	97	99
2	[DBUH][OAc]	88	90	93
3	[DBUH][TFE]	46	63	62
4	[DBUH][TFA]	54	55	54
5	[DBUH]Cl	75	50	50
6	[TBDH][Im]	75	57	54
7	[TMGH][Im]	76	49	46
8	[P_4444_][Im]	34	44	43
9	[TBDH][TFE]	43	99	99

^[a]^ Reaction conditions: **1a** (302.4 mg, 2.0 mmol), **2a** (168.2 mg, 2.0 mmol), IL (47.8 mg, 10%), CO_2_ (1 atm), 80 °C, 12 h. ^[b]^ Determined by ^1^H-NMR with 1,1,2,2-tetrachloroethane as the internal standard.

## Supplementary Materials

The following are available online, Figure S1: ^1^H NMR for propargyl alcohol and mixtures of propargyl alcohol and 2-aminoalcohol.
