# Supplementary material for: Ionic Liquid-Promoted Three-Component Domino Reaction of Propargyl Alcohols, Carbon Dioxide and 2-Aminoethanols: A Thermodynamically Favorable Synthesis of 2-Oxazolidinones
Source: Molecules. 2018 Nov 20;23(11):3033. doi: 10.3390/molecules23113033 (PMC6280151; doi:10.3390/molecules23113033)
Supplement: Supplementary file 1 [file molecules-23-03033-s001.pdf]

molecules

MDPI

Supplementary Materials

Ionic liquid-promoted three-component domino reaction of propargyl alcohols, carbon dioxide and 2-aminoethanols: A thermodynamically favorable synthesis of 2-oxazolidinones

Shu-Mei Xia [1], Yu Song [1], Xue-Dong Li [1], Hong-Ru Li*[1,2], Liang-Nian He[1],*

[1] State Key Laboratory and Institute of Elemento-Organic Chemistry, College of Chemistry, Nankai University, Tianjin, 300071, P. R. China.

[2] College of Pharmacy, Nankai University, Tianjin 300353, P. R. China

Characterization Data for Substrates and Products

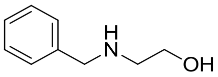

1a

2-(Benzylamino)ethanol[1]. Colorless oil. ^{1}H NMR (400 MHz, $CDCl_3$) δ 7.37-7.27 (m, 5H), 3.80 (s, 2H), 3.66 (t, *J*= 6.0 Hz, 2H), 2.79 (t, *J*= 6.0 Hz, 2H), 2.71 (-OH, -NH) ppm. ^{13}C NMR (100.6 MHz, $CDCl_3$) δ 139.6, 128.4, 128.1, 127.1, 60.7, 53.4, 50.5 ppm. GC-MS (EI, 70 eV) m/z (%) 120.15 (48.51), 91.15 (100).

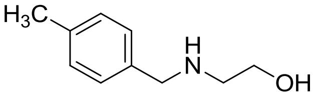

1b

2-(4-Methylbenzylamino)ethanol[1]. Colorless oil. ^{1}H NMR (400 MHz, $CDCl_3$) δ 7.20-7.12 (m, 4H), 3.74 (s, 2H), 3.63 (t, *J*= 6.0 Hz, 2H), 2.75 (t, *J*= 6.0 Hz, 2H), 2.67 (-OH, -NH), 2.33 (s, 3H) ppm. ^{13}C NMR (100.6 MHz, $CDCl_3$) δ 136.7, 136.6, 129.1, 128.1, 60.7, 53.2, 50.5, 21.0 ppm. GC-MS (EI, 70 eV) m/z (%) 134.15 (35.23), 105.10 (100), 77.05 (8.65).

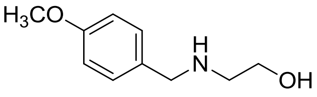

1c

2-(4-Methoxybenzylamino)ethanol[1]. Light yellow oil. ^{1}H NMR (400 MHz, $CDCl_3$) δ 7.21-7.20 (m, 2H), 6.86-6.84 (m, 2H), 3.78 (s, 3H), 3.70 (t, *J*= 6.0 Hz, 2H), 3.62 (t, *J*= 6.0 Hz, 2H), 2.73 (4H) ppm. ^{13}C NMR (100.6 MHz, $CDCl_3$) δ 158.6, 131.9, 129.3, 113.7, 60.7, 55.2, 52.9, 50.5 ppm. GC-MS (EI, 70 eV) m/z (%) 150.20 (17.75), 122.10 (9.08), 121.15 (100), 91.10 (5.24), 78.10 (6.03), 77.05 (7.48).

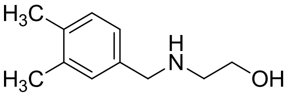

1d

2-(3,4-Dimethylbenzylbenzylamino)ethanol[1]. Colorless solid. M.P. 43 °C. ^{1}H NMR (400 MHz, $CDCl_3$) δ 7.10-7.03 (m, 3H), 3.73 (s, *J*= 6.0 Hz, 2H), 3.65 (t, *J*= 6.0 Hz, 2H), 2.78 (t, 2H), 2.72 (-OH, -NH), 2.26-2.25 (m, 6H) ppm. ^{13}C NMR (100.6 MHz, $CDCl_3$) δ 137.1, 136.7, 135.4, 129.7, 129.6, 125.7, 60.8, 53.2, 50.5, 19.7, 19.4 ppm. GC-MS (EI, 70 eV) m/z (%) 148.20 (26.96), 120.15 (10.86), 119.15 (100), 91.10 (11.06), 77.10 (5.90).

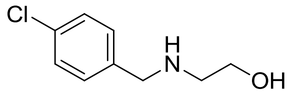

1e

2-(4-Chlorobenzylbenzylamino)ethanol[1]. Light yellow oil. ^{1}H NMR (400 MHz, $CDCl_3$) δ 7.31-7.24 (m, 4H), 3.77 (s, 2H), 3.66 (t, *J*= 6.0 Hz, 2H), 2.78 (t, *J*= 6.0 Hz, 2H), 2.40 (-OH, -NH) ppm. ^{13}C NMR (100.6 MHz, $CDCl_3$) δ 138.2, 132.9, 129.5, 128.6, 60.8, 52.7, 50.4 ppm. GC-MS (EI, 70 eV) m/z (%) 156.10 (9.60), 154.10 (30.14), 127.10 (32.04), 126.10 (7.91), 125.10 (100), 89.05 (14.34).

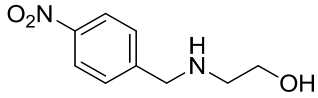

1f

2-(4-Nitrobenzylbenzylamino)ethanol[1]. Brown solid. M.P. 82-83.5 °C. ^{1}H NMR (400 MHz, $CDCl_3$) δ 8.17-8.15 (m, 2H), 7.50-7.48 (m, 2H), 3.91 (s, 2H), 3.68 (t, *J*= 6.0 Hz, 2H), 2.79 (t, *J*= 6.0 Hz, 2H), 2.16 (-OH, -NH) ppm. ^{13}C NMR (100.6 MHz, $CDCl_3$) δ 147.7, 147.0, 128.6, 123.6, 61.0, 52.7, 50.6 ppm. GC-MS (EI, 70 eV) m/z (%) 166.10 (10.39), 165.10 (100), 137.10 (5.32), 136.10 (62.21), 120.10 (8.87), 119.10 (7.69), 106.10 (31.23), 105.05 (5.28), 91.10 (10.32), 90.10 (24.52), 89.05 (24.99), 78.05 (33.75), 77.05 (7.24).

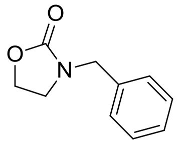

3a

3-Benzyloxazolidin-2-one[2]. Light yellow solid. M.P. 77-78.5 °C. ^{1}H NMR (400 MHz, $CDCl_3$) δ 7.38-7.28 (m, 5H), 4.43 (s, 2H), 4.30 (t, *J* = 8.0 Hz, 2H), 3.42 (t, *J* = 8.0 Hz, 2H) ppm. ^{13}C NMR (100.6 MHz, $CDCl_3$) δ 158.5 (C=O), 135.7, 128.7, 128.0, 127.9, 61.7, 48.3, 43.9 ppm. GC-MS (EI, 70 eV) m/z (%) 178.10 (7.73), 177.10 (62.67), 176.10 (61.59), 132.15 (19.98), 105.10 (27.09), 104.10 (100), 92.10 (14.18), 91.10 (86.07), 78.10 (18.29), 77.10 (11.92), 65.10 (27.49).

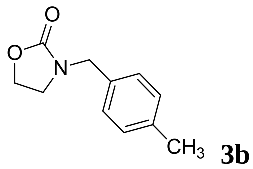

3b

3-(4-Methylbenzyl)oxazolidin-2-one[3]. Colorless oil. ^{1}H NMR (400 MHz, $CDCl_3$) δ 7.19-7.14 (m, 4H), 4.39 (s, 2H), 4.28 (t, *J* = 8.0 Hz, 2H), 3.42 (t, *J* = 8.8 Hz 2H), 2.34 (s, 3H) ppm. ^{13}C NMR (100.6 MHz, $CDCl_3$) δ 158.5 (C=O), 137.7, 132.7, 129.5, 128.2, 61.7, 48.1, 43.8, 21.1 ppm. GC-MS (EI, 70 eV) m/z (%) 191.20 (48.54), 176.20 (58.84), 146.25 (7.91), 132.20 (15.10), 119.15 (24.23), 118.15 (100), 105.15 (60.83), 91.10 (23.07), 77.10 (25.82).

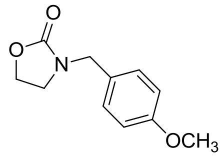
3c

3-(4-Methoxybenzyl)oxazolidin-2-one[2]. Colorless solid. M.P. 72-73.5 °C. ^{1}H NMR (400 MHz, $CDCl_3$) δ 7.22-7.20 (m, 2H), 6.88-6.86 (2H), 4.36 (s, 2H), 4.28 (t, J = 7.2 Hz, 2H), 3.80 (s, 3H), 3.39 (t, J = 7.6 Hz, 2H) ppm. ^{13}C NMR (100.6 MHz, $CDCl_3$) δ 159.3, 158.4, 129.5, 127.8, 114.1, 61.7, 55.3, 47.8, 43.8 ppm. GC-MS (EI, 70 eV) m/z (%) 208.20 (6.69), 207.20 (48.54), 206.20 (26.48), 179.15 (20.05), 176.20 (29.66), 162.20 (9.17), 135.15 (23.28), 134.20 (100), 121.15 (68.48), 91.10 (12.46), 78.10 (20.27), 77.10 (20.87), 65.10 (8.26), 63.05 (5.09).

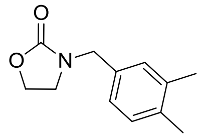
3d

3-(3,4-Dimethylbenzyl)oxazolidin-2-one[1]. Light yellow oil. ^{1}H NMR (400 MHz, $CDCl_3$) δ 7.11-6.99 (m, 3H), 4.35 (s, 2H), 4.27 (t, J = 8.0 Hz, 2H), 3.40 (t, J = 8.0 Hz, 2H), 2.25 (s, 6H) ppm. ^{13}C NMR (100.6 MHz, $CDCl_3$) δ 158.4 (C=O), 136.9, 136.1, 132.9, 129.8, 129.3, 125.5, 61.6, 47.9, 43.7, 19.5, 19.2 ppm. GC-MS (EI, 70 eV) m/z (%) 146.20 (24.14), 133.20 (25.43), 132.20 (100), 119.20 (72.16), 106.15 (12.16), 105.15 (18.44), 104.15 (8.77), 91.10 (42.09), 77.10 (24.38), 65.10 (10.50). HRMS (ESI): $C_{12}H_{16}NO_2$ for $[M+H]^+$ calculated 206.1176, found 206.1181.

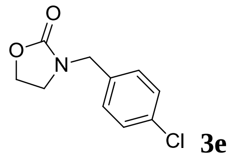
3e

3-(4-Chlorobenzyl)oxazolidin-2-one[1]. M.P. 72-73 °C. ^{1}H NMR (400 MHz, $CDCl_3$) δ 7.32-7.30 (m, 2H), 7.22-7.20 (m, 2H), 4.38 (s, 2H), 4.29 (t, J = 8.0 Hz, 2H), 3.40 (t, J = 8.0 Hz, 2H) ppm. ^{13}C NMR (100.6 MHz, $CDCl_3$) δ 158.4 (C=O), 134.2, 133.8, 129.4, 128.9, 61.7, 47.7, 43.9 ppm. GC-MS (EI, 70 eV) m/z (%) 213.15 (14.97), 211.15 (47.48), 210.15 (14.92), 176.15 (52.54), 166.15 (9.45), 138.15 (100), 132.20 (25.87), 125.10 (72.60), 112.10 (12.73), 89.10 (37.59), 77.10 (12.46), 63.00 (15.51).

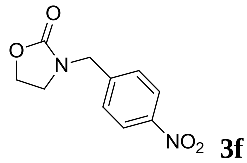
3f

3-(4-Nitrobenzyl)oxazolidin-2-one[2]. Light yellow solid. M.P. 148-150 °C. ^{1}H NMR (400 MHz, $CDCl_3$) δ 7.34-7.22 (4H), 4.40 (s, 2H), 4.32 (t, J = 8.0 Hz, 2H), 3.43 (t, J = 8.0 Hz, 2H) ppm. ^{13}C NMR (100.6 MHz, $CDCl_3$) δ 158.4 (C=O), 134.2, 133.8, 129.4, 128.9, 61.7, 47.6, 43.8 ppm. GC-MS (EI, 70 eV) m/z (%) 213.15 (16.79), 212.10 (10.73), 211.10 (49.09), 210.10 (15.33), 177.25 (6.14), 176.15 (51.73), 166.15 (9.83), 140.10 (35.32), 139.10 (25.34), 138.10 (100), 132.20 (26.26), 127.10 (22.41), 126.10 (7.12), 125.10 (73.59), 112.10 (12.93), 89.10 (35.12), 77.10 (12.20), 76.10 (8.47), 63.05 (14.85).

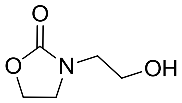
3g

3-(2-Hydroxyethyl)oxazolidin-2-one. Colorless oil. ^{1}H NMR ($CDCl_3$, 400 MHz) δ 4.32 (t, J = 8.0 Hz, 2H), 3.73-3.62 (m, 5H), 3.31 (t, J = 5.0 Hz, 2H) ppm. ^{13}C NMR (100.6 MHz, $CDCl_3$) δ 159.2 (C=O), 62.1, 59.8, 46.5, 45.3 ppm. GC-MS (EI, 70 eV) m/z (%) 113.10 (7.88), 101.10 (66.73), 100.10 (100), 88.10 (12.34).

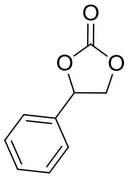
3h

4-Phenyl-1,3-dioxolan-2-one[4]. White solid. M.P. 53 °C; ^{1}H NMR (400 MHz, $CDCl_3$) δ 4.32 (t, J = 8.4Hz, 1H), 4.78 (t, J = 8.4 Hz, 1H), 5.70 (t, J = 8.0 Hz, 1H), 7.35 (d, J = 7.6 Hz, 2H), 7.43 (d, J = 6.4 Hz, 3H). ^{13}C NMR (100.6 MHz, $CDCl_3$) δ 71.29, 78.11, 125.99, 129.34, 129.84, 135.86, 154.97. GC-MS (EI, 70 eV) m/z (%) 164.10 (69), 120.10 (13), 119.10 (12), 105.10 (31), 92.10 (20), 91.10 (96), 90.05 (100), 89.05 (36), 78.10 (78), 77.05 (28), 65.05 (27), 63.05 (14).

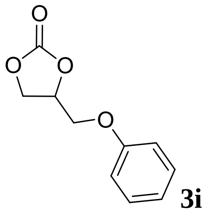
3i

4-(Phenoxymethyl)-1,3-dioxolan-2-one[4]. Yellow liquid. ^{1}H NMR (400 MHz, $CDCl_3$) δ 4.14 (dd, ^3J= 4.4 Hz, ^2J = 10.8 Hz, 1H), 4.23 (dd, ^3J = 3.6 Hz, ^2J = 10.8 Hz, 1H) 4.54 (dd, ^3J = 8.4 Hz, ^2J= 6.0 Hz, 1H), 4.60 (t, J = 8.4 Hz, 1H), 5.02 (m, 1H), 6.90 (d, J = 8.0 Hz, 2H), 7.00 (t, J = 7.4 Hz, 2H), 7.31 (t, J = 8.0 Hz, 2H); ^{13}C NMR (100.6 MHz, $CDCl_3$) δ 44.92, 67.02, 74.43, 114.8, 122.2, 129.1, 154.39. GC-MS (EI, 70 eV) m/z (%) 194.05 (66), 107.10 (100), 94.05 (73), 77.10 (87), 65.05 (18), 51.05 (23), 43.05 (12).

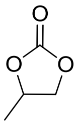
3j

4-Methyl-1,3-dioxolan-2-one. Colorless liquid[4]. ^{1}H NMR (400 MHz, $CDCl_3$) δ 1.39 (d, J = 6.0 Hz, 1H), 3.96 (t, 1H), 4.49 (t, J = 8.4 Hz, 1H), 4.79 (m, 1H). ^{13}C NMR (100.6 MHz, $CDCl_3$) δ 19.28, 70.72, 73.71, 155.16. GC-MS (EI, 70 eV) m/z (%) 102.05 (19), 87.05 (100).

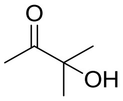
4a

3-Hydroxy-3-methylbutan-2-one[4]. Colorless oil. ^{1}H NMR (400 MHz, DMSO-d_6) δ 5.24 (s, 1H), 2.15 (s, 3H), 1.17 (s, 6H) ppm. ^{13}C NMR (100.6 MHz, DMSO-d_6) δ 213.6, 75.5, 25.9, 24.0 ppm. GC-MS (EI, 70 eV) m/z (%) 102.10 (8.57), 87.10 (100), 69.05 (61.60), 60.05 (92.12).

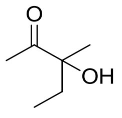

4b

3-Hydroxy-3-methylpentan-2-one[4]. Colorless oil. ^{1}H NMR (400 MHz, DMSO-d_6) δ 5.04 (OH, 1H), 2.13 (s, 3H), 1.64-1.42 (m, 2H), 1.12 (s, 3H), 0.74 (t, J = 7.4 Hz, 3H) ppm. ^{13}C NMR (100.6 MHz, DMSO-d_6) δ 214.2, 78.6, 31.8, 25.1, 24.1, 7.9 ppm. GC-MS (EI, 70 eV) m/z (%) 67.10 (100), 85.05 (66.74), 71.10 (15.76), 69.10 (12.80), 84.10 (12.44), 86.10 (12.00), 110.10 (10.92), 95.10 (10.56).

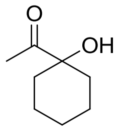

4c

1-(1-Hydroxycyclohexyl)ethanone[4]. Yellow oil. ^{1}H NMR (400 MHz, $CDCl_3$) δ 2.24 (s, 3H), 1.75-1.64 (m, 6H), 1.49 (d, J = 6.5 Hz, 2H), 1.28 (dd, J_1 = 15.1 Hz, J_2 = 10.3 Hz, 2H) ppm. ^{13}C NMR (100.6 MHz, $CDCl_3$) δ 212.7, 78.0, 33.8, 25.3, 23.7, 21.1 ppm. GC-MS (EI, 70 eV) *m/z* (%) 99.10 (70.66), 81.10 (100), 79.10 (20.54).

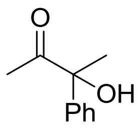

4d

3-Hydroxy-3-phenylbutan-2-one[4]. Brown oil. ^{1}H NMR (400 MHz, DMSO-d_6) δ 7.43 (d, J = 7.6 Hz, 2H), 7.35 (t, J = 7.4 Hz, 2H), 7.26 (t, J = 7.1 Hz, 1H), 6.06 (s, 1H), 2.02 (s, 3H), 1.52 (s, 3H) ppm. ^{13}C NMR (100.6 MHz, DMSO-d_6) δ 210.0, 143.0, 127.9, 126.9, 124.7, 79.4, 25.8, 24.0 ppm. GC-MS (EI, 70 eV) *m/z* (%) 121.10 (100), 105.10 (18.88), 77.05 (30.87).

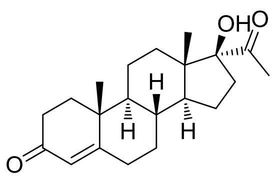

4e

White solid[1], M. p. 192–193 °C.^{1}H NMR (400 MHz, $CDCl_3$) δ 5.69 (s, 1H), 2.96 (s, 1H), 2.38-2.27 (m, 5H), 2.23 (s, 3H), 1.98-1.95 (m, 1H), 1.86-1.83 (m, 1H), 1.74-1.36 (m, 10H), 1.16 (s, 3H), 1.06-1.02 (m, 1H), 0.95 (s, 3H), 0.90-0.82 (m, 1H) ppm. ^{13}C NMR (100.6 MHz, $CDCl_3$) δ 214.2, 199.4, 171.0, 123.8, 90.7, 53.2, 49.1, 47.5, 38.5, 36.1, 35.6, 35.0, 33.8, 33.0, 32.7, 31.5, 28.2, 24.2, 20.7, 17.3, 14.1 ppm. HRMS (ESI): $C_{21}H_{31}O_3$ for $[M+H]^+$ calculated 331.2268, found 331.2274

References

1. Li, X.D.; Cao, Y.; Ma, R.; He, L.N. Thermodynamically favorable protocol for the synthesis of 2-oxazolidinones via Cu(I)-catalyzed three-component reaction of propargylic alcohols, CO_2 and 2-aminoethanols. *J. CO_2 Util.* **2018,** *25*, 338-345, DOI:10.1016/j.jcou.2018.01.022.
2. Buyck, T.; Pasche, D.; Wang, Q.; Zhu, J. Synthesis of Oxazolidin-2-ones by Oxidative Coupling of Isonitriles, Phenyl Vinyl Selenone and Water. *Chem.* **2016, 22**, 2278-2281, DOI: 10.1002/chem.201505050.
3. Ravikumar, K.S.; Kesavan, V.; Crousse, B.; Bonnet-Delpon, D.; Bégué, J.P. Mild and Selective Oxidation of Sulfur Compounds in Trifluoroethanol: Diphenyl Disulfide and Methyl Phenyl Sulfoxide. *Org. Synth.* **2003**, *80*, 184-189, DOI: 10.15227/orgsyn.080.0184.
4. Ying, A.G.; Chen, X.Z.; Ye, W.D.; Chen, G.; Chen, X.Z.; Ye, W.D. Aza-Michael addition of aliphatic or aromatic amines to α,β-unsaturated compounds catalyzed by a DBU-derived ionic liquid under solvent-free conditions. *Tetrahedron Lett.* **2009,** *50*, 1653-1657, DOI: 10.1016/j.tetlet.2009.01.123
